# Measuring Myeloperoxidase Activity in Biological Samples

**DOI:** 10.1371/journal.pone.0067976

**Published:** 2013-07-05

**Authors:** Benjamin Pulli, Muhammad Ali, Reza Forghani, Stefan Schob, Kevin L. C. Hsieh, Gregory Wojtkiewicz, Jenny J. Linnoila, John W. Chen

**Affiliations:** 1 Center for Systems Biology, Massachusetts General Hospital and Harvard Medical School, Boston, Massachusetts, United States of America; 2 Department of Radiology, Massachusetts General Hospital, Boston, Massachusetts, United States of America; 3 Department of Radiology, Jewish General Hospital and McGill University, Montreal, Canada; 4 Department of Medical Imaging, Far-Eastern Memorial Hospital, Taipei, Taiwan; University of Kansas Medical Center, United States of America

## Abstract

**Background:**

Enzymatic activity measurements of the highly oxidative enzyme myeloperoxidase (MPO), which is implicated in many diseases, are widely used in the literature, but often suffer from nonspecificity and lack of uniformity. Thus, validation and standardization are needed to establish a robust method that is highly specific, sensitive, and reproducible for assaying MPO activity in biological samples.

**Principal findings:**

We found conflicting results between *in vivo* molecular MR imaging of MPO, which measures extracellular activity, and commonly used *in vitro* MPO activity assays. Thus, we established and validated a protocol to obtain extra- and intracellular MPO from murine organs. To validate the MPO activity assays, three different classes of MPO activity assays were used in spike and recovery experiments. However, these assay methods yielded inconsistent results, likely because of interfering substances and other peroxidases present in tissue extracts. To circumvent this, we first captured MPO with an antibody. The MPO activity of the resultant samples was assessed by ADHP and validated against samples from MPO-knockout mice in murine disease models of multiple sclerosis, steatohepatitis, and myocardial infarction. We found the measurements performed using this protocol to be highly specific and reproducible, and when performed using ADHP, to be highly sensitive over a broad range. In addition, we found that intracellular MPO activity correlated well with tissue neutrophil content, and can be used as a marker to assess neutrophil infiltration in the tissue.

**Conclusion:**

We validated a highly specific and sensitive assay protocol that should be used as the standard method for all MPO activity assays in biological samples. We also established a method to obtain extra- and intracellular MPO from murine organs. Extracellular MPO activity gives an estimate of the oxidative stress in inflammatory diseases, while intracellular MPO activity correlates well with tissue neutrophil content. A detailed step-by-step protocol is provided.

## Introduction

Myeloperoxidase (MPO) is the most abundant proinflammatory enzyme stored in the azurophilic granules of neutrophilic granulocytes, accounting for approximately 5% of their dry mass [1]. It catalyzes the formation of hypochlorous acid from hydrogen peroxide, generates other highly reactive molecules such as tyrosyl radicals, and cross-links proteins [2]. Recently, MPO has been found to be implicated in a multitude of diseases, including atherosclerosis [3], myocardial infarction [4], [5], atrial fibrillation [6], multiple sclerosis [7], Alzheimer’s disease [8], lung cancer [9], and transplant rejection [10]. Scientific research on MPO has steadily increased over the last 2 decades, with approximately 1000 manuscripts published in 2012 alone.

While MPO expression or protein level measurements can provide some information regarding the abundance of the MPO molecule, the enzymatic activity of MPO can vary considerably between individuals even if the amount of MPO present is similar [11]. Besides effects such as age and gender, multiple polymorphisms have been identified both with decreased [12] and increased [11] MPO activity. Furthermore, as MPO can be inhibited by endogenous inhibitors, MPO activity does not always correspond to MPO protein or expression levels [13], [14].

Evaluating MPO activity is crucial to understanding its effects in inflammation, and it is not surprising that MPO activity assays are widely used in the literature for this purpose. However, no consensus has been reached on which of the many available assays to use. This is further complicated by the fact that most available probes (e.g. TMB, *o*-dianisidine, guaiacol) are general peroxidase substrates, lacking specificity towards MPO. Moreover, tissue inhibitors of MPO can interfere with assays [15], and peroxidase activity of myoglobin and hemoglobin can also alter assay results [16]. More importantly, different assays have not yet been compared to each other, and validation and standardization are much needed to verify results from different studies.

In this work we compared different peroxidation and chlorination assays of MPO for their specificity and utility in evaluating biological samples. We established a method to isolate extracellular as well as intracellular MPO, and measured activity with high specificity and reproducibility using an antibody capture activity assay. We provide a detailed protocol (Protocol S1 in File S2) validated against MPO-knockout mice on multiple murine organs and different disease models, and propose this assay protocol be used as the standard method to measure MPO activity in biological samples.

## Materials and Methods

All protocols for animal experiments were approved by the Institutional Animal Care and Use Committee (IACUC) at the Massachusetts General Hospital, Boston, MA.

### Literature Search

To evaluate the choice of MPO activity assays over the past several years, scientific manuscripts were searched for in PubMed using the search string ‘myeloperoxidase activity’ OR ‘MPO activity’. Work between January 1, 2011 and December 31, 2012 was considered. Manuscripts were then evaluated for the use of MPO activity assays and the specific tissues and methods used.

### Animal Models

All procedures were performed under anesthesia with 1–3% isoflurane (Forane, Baxter, Deerfield, IL). C57Bl/6J wildtype (WT) and MPO knockout (KO) mice (Jackson laboratories, Bar Harbor, ME) were used.

The permanent coronary ligation model was used to induce myocardial infarction (MI) in age-matched male WT and MPO-KO mice as described previously [17]. Briefly, anesthetized mice were intubated, and mechanically ventilated with a single animal pressure/volume controlled ventilator (Harvard Apparatus, Holliston, MA). A thoracotomy was performed in the left fourth intercostal space, and the left coronary artery was permanently ligated. At day 2 post surgery, plasma and heart were extracted for further tissue processing.

Experimental autoimmune encephalomyelitis (EAE) was induced in age-matched female WT and MPO-KO mice 6–10 weeks old with myelin oligodendrocyte glycoprotein (MOG_35–55_, Neo Bioscience, Cambridge, MA) [18]. Briefly, each mouse was injected subcutaneously at 4 sites (bilateral inguinal and axillary) with a suspension containing 800 µg M. tuberculosis H37RA (BD Difco, Franklin Lakes, NJ), 200 µg MOG_35–55_ in one part complete Freund’s adjuvant (CFA, Sigma, St. Louis, MO) mixed with one part phosphate buffered saline (PBS). Within two hours of induction and on day 2, mice received 0.25 µg pertussis toxin (Sigma) intravenously. For experiments involving *in-vivo* MR imaging with MPO-Gd, female SJL mice (NCI, Frederick, MD) were induced with 100 µg proteolipid protein (PLP_139–151_) in one part CFA mixed with one part PBS containing 400 µg M. tuberculosis H37RA. Within two hours of induction and on day 2, SJL mice received 0.1 µg pertussis toxin (Sigma) intravenously. Animals were sacrificed at the peak of disease (days 14–18 post induction for C57BL/6J and MPO-knockout mice, and days 10–13 post induction for SJL mice), and brains were harvested for analysis.

For induction of non-alcoholic steatohepatitis (NASH), age-matched female WT and MPO-KO were fed a diet deficient in methionine and choline (MCD diet, Harlan, Indianapolis, IN) for 4 weeks. Dietary methionine and choline are required for hepatic beta-oxidation and VLDL-secretion, and their absence triggers steatohepatitis with increased oxidative stress, proinflammatory cytokines, and histological findings resembling human NASH [19].

### *In vivo* MPO-Gd Molecular MR Imaging

To confirm the presence of MPO activity in EAE *in vivo* and to evaluate the effects of the specific MPO inhibitor ABAH [20] (4-Aminobenzoic acid hydrazide, Sigma) noninvasively, we performed MPO-Gd (*bis*-5-hydroxytryptamide-diethylenetriaminepentaacetate-gadolinium) molecular MR imaging in mice with EAE. MPO-Gd is an activatable MR imaging agent that reports *extracellular* MPO activity *in vivo* with high specificity and sensitivity [21], [22]. It was synthesized in our laboratory as previously described [22]. MR imaging was performed by using an animal 4.7-T MR imaging unit (Bruker, Billerica, MA) before and after intravenous administration of MPO-Gd (0.3 mmol/kg). *In vivo* MPO activation was determined by calculating the activation ratio (ratio of contrast-to-noise ratios [CNRs] of lesions at delayed [60 minutes] and early [15 minutes] time points) [23]. Early enhancement represents mostly leakage through blood-brain barrier breakdown, whereas delayed enhancement is derived mostly from agent retention caused by MPO activation.

### Tissue Processing and Protein Precipitation

Anesthetized mice were transcardially perfused with 20 mL PBS to clear the intravascular compartment of blood cells. For analysis of whole tissue MPO activity, brains were harvested and homogenized by a mechanical homogenizer (Tissuemiser, Fisher Scientific, Waltham, MA) in 500 µl CTAB buffer (50 mM cetyltrimethylammonium bromide [CTAB, Sigma] in 50 mM potassium phosphate buffer at pH = 6), sonicated, and centrifuged at 15,000 g for 20 min. The supernatant was used for protein analysis with a BCA protein assay kit (Thermo Scientific, Waltham, MA) and for MPO activity assays. For separation of extra- (ECF) and intracellular protein fractions (ICF) we modified a method initially described for mouse brains [24]. We washed harvested organs (kidney, brain, liver, heart, spleen, and lungs) three times in PBS and incubated them for 2 hours in extraction buffer (0.32 M sucrose [Sigma], 1 mM CaCl_2_ [Sigma], 10 U/ml Heparin [APP Pharmaceuticals, Schaumburg, IL] in Hanks Balanced Salt Solution [HBSS]). Then, organs were removed from the solution and processed in the same way as for whole tissue MPO activity to obtain the ICF. The extraction buffer containing the ECF was then centrifuged at 1000 g for 5 min to pellet any cellular debris, and the supernatant underwent protein precipitation by slow mixing with 4 parts ice-cold acetone (Fisher Scientific). This was performed in order to concentrate the very dilute extracellular fraction. The acetone-protein mixture was then incubated for 1 hour at −20°C, and proteins were precipitated by centrifugation at 3500 g for 15 min at 4°C. The supernatant was discarded, and the protein pellet was air-dried and resuspended in PBS for BCA and MPO activity assays.

Optimal protein precipitation conditions for MPO were tested by using purified human MPO (1.7 mg/ml; Lee Biosolutions, St. Louis, MO). 0.24, 0.12, 0.06 and 0.03 pmol MPO were precipitated with either acetone or ammonium sulfate as previously described [25]. Recovery of MPO after precipitation was compared to unprecipitated MPO activity using 10-acetyl-3,7-dihydroxyphenoxazine (ADHP, AAT Bioquest, Sunnyvale, CA) as described below.

### MPO Activity Assays

All activity assays were performed in duplicates or triplicates on 96 well microtiter plates and analyzed with a Safire 2 microplate reader (Tecan, Durham, NC).

Peroxidase activity with 3,3′,5,5′-Tetramethylbenzidine (TMB, Sigma) was measured as described before [26]. Briefly, 10 µl sample were combined with 80 µl 0.75 mM H_2_O_2_ (Sigma) and 110 µl TMB solution (2.9 mM TMB in 14.5% DMSO [Sigma] and 150 mM sodium phosphate buffer at pH 5.4), and the plate was incubated at 37°C for 5 min. The reaction was stopped by adding 50 µl 2 M H_2_SO_4_ (Sigma), and absorption was measured at 450 nm to estimate MPO activity.

Peroxidase activity was also assessed with 10-acetyl-3,7-dihydroxyphenoxazine (ADHP, AAT Bioquest, Sunnyvale, CA) at an excitation wavelength of 535 nm and an emission wavelength of 590 nm. Briefly, 10 µl sample were combined with 39 µl PBS and 1 µl of 1∶100 diluted 3% hydrogen peroxide (H_2_O_2_), then 50 µl of 200 µM ADHP solution was added. The assays were performed immediately and data was collected from a total of 50 kinetic cycles (Fig. S1 in File S1). Enzyme activity was defined as the slope of the data.

Chlorination activity of MPO was evaluated with 3′-(p-aminophenyl) fluorescein (APF) and 3′-(p-hydroxyphenyl) fluorescein (HPF, both from Cayman Chemicals, Ann Arbor, MI) [27]. 20 µl sample were combined with 40 µl 2 µM H_2_O_2_ and 40 µl 20 µM APF or HPF (in 1% methyl acetate). Fluorescence was measured at 499 nm excitation and 515 nm emission after 30 minutes. HPF fluorescence values were subtracted from APF in order to calculate MPO chlorination activity.

Bromide-dependent chemiluminescence with luminol (Sigma) was measured as previously described [28]. Briefly, 10 µl sample were combined with 30 µl of 33.3 mM H_2_O_2_ and 40 µl of 25 µM luminol, both in 0.1 M sodium acetate buffer (pH = 5.0). Chemiluminescence was then measured for 3 minutes, after which 20 µl 20 mM potassium bromide (Br, Sigma) was added to each well, and chemiluminescence was measured for 3 minutes. MPO activity was measured by subtraction of Br-dependent signal from the first 3 minutes of readout. At the acidic pH of 5, this has been suggested to result in specific MPO activity detection [29].

To specifically capture MPO, samples were incubated in MPO ELISA dilution buffer (Hycult, Plymouth Meeting, PA) on anti-MPO antibody coated plates (Hycult) for 1 hour at room temperature. Assay wells were then washed 4 times with washing buffer (PBS with 0.05% Tween 20), and once with PBS only. MPO activity of antibody-captured MPO was assessed with ADHP as above.

### Spike and Recovery Assay

To test recovery of MPO from different organ extracts, pure human MPO from neutrophil extracts (Lee Biosolutions) was used as positive control at either 0.12 pmol or 0.05 pmol, and added to ICF and ECF of isolated organs. MPO activity assays (with ADHP, luminol, and APF/HPF, as described above) were then performed to investigate recovery of MPO activity from organ supernatants, and normalized as a percentage of MPO positive control.

### LDH Assay

To confirm successful isolation of ECF and ICF, we conducted an LDH assay (Cayman Chemicals) as per manufacturer’s instructions. LDH is a strictly intracellular enzyme, and its presence outside of cells indicates cell death. Briefly, samples were diluted in assay buffer and combined with NAD^+^, lactic acid, tetrazolium salt, and diaphorase, and absorption was read at 490 nm. LDH activity in µU was normalized to BCA protein assay concentration, and the ratio of intra- to extracellular LDH was reported.

### Neutrophil Isolation

Neutrophils were isolated using an established protocol [30]. Briefly, femur and tibia bone shafts were flushed with staining buffer (Dulbecco’s phosphate buffered saline [DPBS] with 0.5% bovine serum albumin [BSA] and 1% fetal bovine serum [FBS]) with a 25 G needle and a 10 ml syringe, filtered through a 40 µm cell strainer (BD Biosciences, San Jose, CA), and centrifuged at 400 g for 7 minutes. Red blood cells were lysed using RBC lysis buffer (Biolegend, San Diego, CA) according to manufacturer’s instructions. Cells were then washed and centrifuged on a 0–62% discontinuous percoll (GE Healthcare, Little Chalfont, UK) gradient for 30 min at 1000 g. The neutrophil-enriched pellet was harvested, and the cells were washed and homogenized by a Tissuemiser homogenizer (Fisher Scientific, Waltham, MA) and sonicated. Cell homogenates underwent three freeze-thaw cycles in liquid nitrogen, and after centrifugation (15,000 g, 20 min) supernatant was used for subsequent experiments as the murine MPO positive control. For the intra-assay reproducibility experiment, 1.2 million cells were serially diluted and loaded in triplicates to simultaneously run a MPO standard curve by using ADHP as explained above. For inter-assay reproducibility, three additional standard curves were run at three different points by using the same samples and dilution factors; each curve was run at least 1 hour apart.

### Flow Cytometry

Organs were harvested as described above. Half of each organ was processed for intra- and extracellular protein fractions, while the other half was used for flow cytometric evaluation of tissue neutrophil content. All organs except heart were dounce homogenized and filtered through a 40 µm cell strainer. Spleen cells were then incubated in RBC lysis buffer as per manufacturer’s instructions. To remove parenchymal liver cells, the cell suspension was centrifuged twice at 50 g for 5 min, and the cell pellet was discarded. The supernatant was then spun at 350 g for 10 min, and liver leukocytes were enriched over a 35% percoll gradient [32]. Brain leukocytes were enriched over a discontinuous 30–70% percoll gradient [33]. Heart tissue was digested with 450 U/ml collagenase I, 125 U/ml collagenase XI, 60 U/ml DNase I and 60 U/ml hyaluronidase [17] (all from Sigma) shaken at 37°C for 1 hour, and then filtered through a 100 µm cell strainer. Cells from all organs were then counted using a hematocytometer, and the cell number of different cell populations was calculated as total cells multiplied by percentage within the respective cell population gate. The following antibodies (all from BD Bioscience, Franklin-Lakes, NJ) were used: anti-Ly-6G, IA8; anti-CD11b, M1/70; and anti-Ly-6C, AL-21. Viable cells were determined by adding 1 µg/ml 4′,6-diamidino-2-phenylindole (DAPI, Invitrogen, Carlsbad, CA) to the cell suspension immediately before analysis, and DAPI-positive cells were excluded. Neutrophils were identified as CD11b^high^, Ly-6G^high^, and Ly-6C^int^.

### Statistical Analysis

Statistical analysis of the data was performed using GraphPad Prism software (GraphPad Software, Inc., La Jolla, CA). Results were expressed as mean ± SD. Statistical tests included Student’s *t-*test using Welch’s correction for unequal variances, and Mann-Whitney U test for non-normal distributed data as determined by the D’Agostino and Pearson omnibus normality test. A *P*-value equal or less than 0.05 was considered statistically significant.

## Results

### Lack of Consensus in the Literature on how to Measure MPO Activity

**Figure 1:**
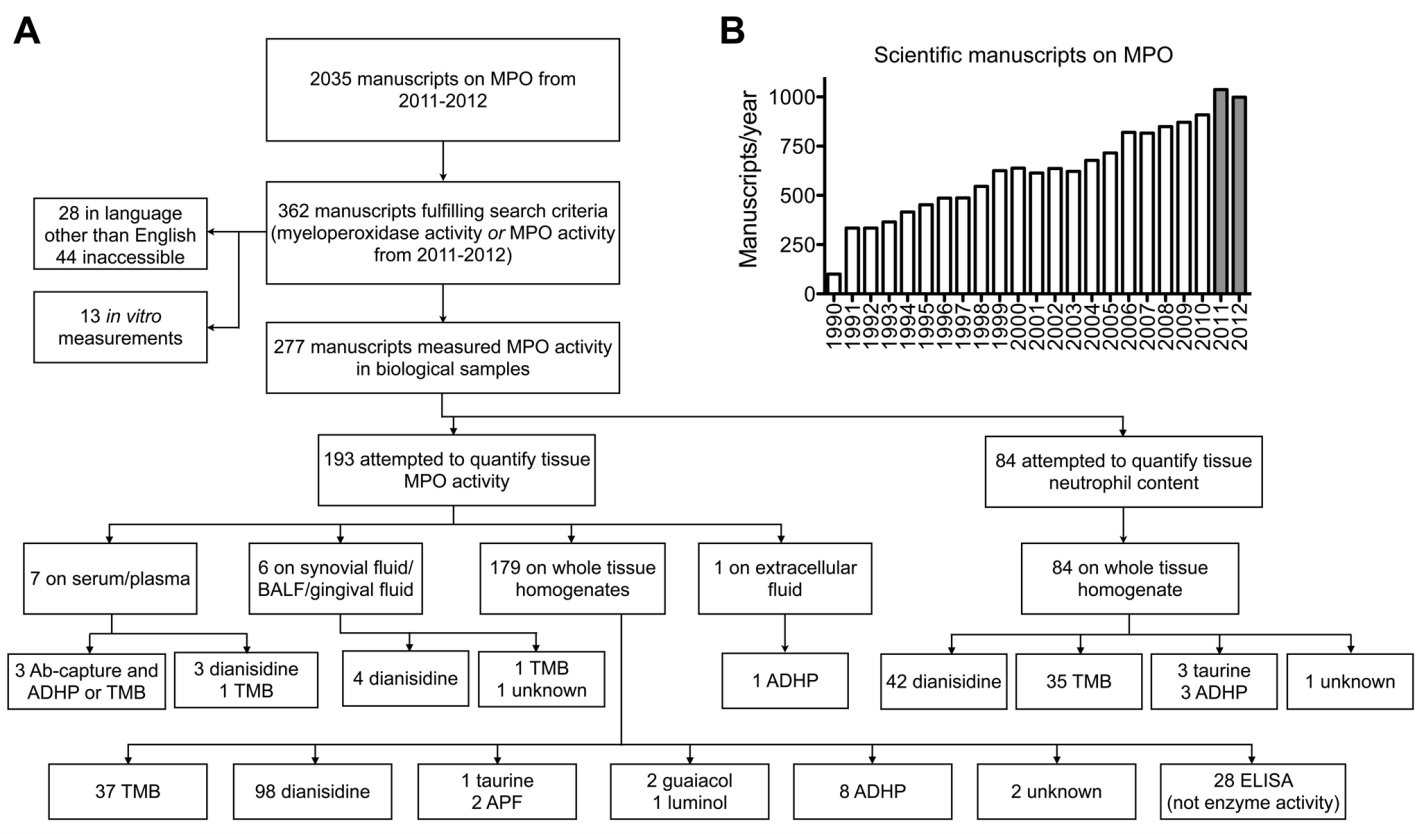
MPO in the literature. (**A**) Usage of MPO activity assays in the Literature from 2011 to 2012. (**B**) Manuscripts published on MPO from 1990 to 2012; grey bars indicate manuscripts considered in (**A**). MPO = myeloperoxidase. TMB = 3,3′,5,5′-Tetramethylbenzidine. ADHP = 10-acetyl-3,7-dihydroxyphenoxazine. BALF = bronchoalveolar lavage fluid. Ab = antibody. APF = 3′-(p-aminophenyl) fluorescein. ELISA = enzyme-linked immunosorbent assay.

Our literature search revealed that out of 2,035 scientific manuscripts published on MPO in 2011 and 2012, 362 assessed for MPO activity. We excluded 28 manuscripts written in languages other than English, and 44 manuscripts where the full text was inaccessible. To further study the methodologies used in these manuscripts, we limited the search to manuscripts that measured MPO activity in tissues rather than cell culture supernatants (n = 13). We found 277 manuscripts (**Fig. 1**). The goal of 193 manuscripts was to evaluate tissue MPO activity, while in 84 manuscripts the authors attempted to quantify tissue neutrophil content. For tissue MPO activity, in most manuscripts whole tissue homogenates were used, and only one paper extracted extracellular fluid. Importantly, we identified 28 papers with the goal to measure MPO activity, but instead of activity assays ELISAs were used, which measure MPO protein content, but not activity. Overall, the most commonly used probes were *o*-dianisidine (n = 147) and TMB (n = 75), followed by ADHP (n = 13), taurine (n = 4), and a few other probes. These results show that there is no consensus in the literature on which assay to use and that incorrect modalities (e.g., ELISA) are also being used. Of note, we did not find work comparing different assays for their ability to specifically detect MPO activity in biological samples.

### *In vivo* MPO Activity Imaging Demonstrates Effect of MPO Inhibition, While *in vitro* MPO Activity Assays do not Detect a Difference

**Figure 2:**
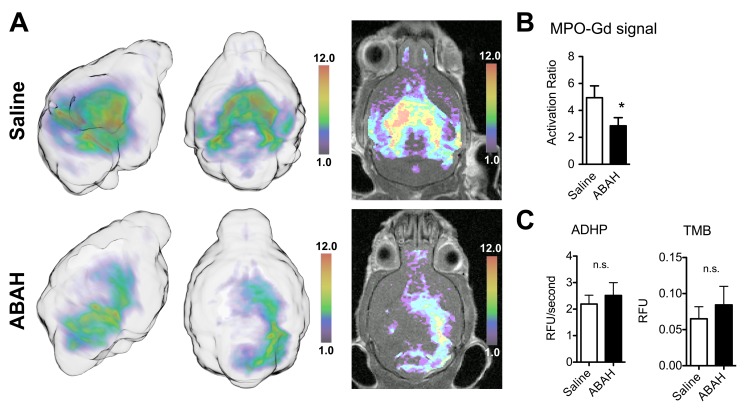
*In vivo* imaging and *in vitro* MPO activity assays demonstrate markedly different findings. (**A**) MPO-Gd molecular MR imaging reveals MPO inhibition in vivo in mice with experimental autoimmune encephalomyelitis that were treated with ABAH. MPO activity maps are shown in 3D from two angles (left), as well as overlays of MPO activity maps over T1 images (right). (**B**) Quantification of imaging reveals significant difference in MPO activity *in vivo* (*P* = 0.03, n = 8 per group). (**C**) *In vitro* assays on whole tissue homogenates using ADHP or TMB do not confirm the *in vivo* imaging finding (*P* = 0.68 and 0.88, respectively, n = 4 per group). *: *P*<0.05, n.s. = not statistically significant. MPO = myeloperoxidase. TMB = 3,3′,5,5′-Tetramethylbenzidine. ADHP = 10-acetyl-3,7-dihydroxyphenoxazine. ABAH = 4-aminobenzoic acid hydrazide. Activation ratio = contrast-to-noise ratio 60 minutes over 15 minutes post MPO-Gd injection.

In the presence of inflammatory stimuli, inflammatory cells degranulate and release their enzymatic contents, such as MPO, into the extracellular environment [34]. When treating EAE mice with ABAH, a specific irreversible MPO inhibitor [20], MPO-Gd MR imaging detected a significant difference between saline control and ABAH-treated mice (**Fig. 2a–b** **,** activation ratio 4.9±0.9 vs. 2.9±0.6; *P* = 0.03). In contrast, MPO activity assays on whole tissue homogenates with ADHP or TMB failed to detect the difference found on imaging (**Fig. 2c**, absorption 0.065±0.034 vs. 0.084±0.051 with TMB, *P* = 0.88; 2.19±0.67 vs. 2.51±0.97 RFU/sec with ADHP, *P* = 0.68).

While MPO-Gd cannot penetrate cells and therefore detects only extracellular MPO activity, our *in vitro* assays were performed on whole tissue homogenates. In addition, ABAH does not inhibit intracellular MPO [35]. While intracellular (stored in granules of neutrophils and in inflammatory monocytes) and extracellular MPO (secreted and/or in neutrophil extracellular traps [NETs]) are implicated in host defense against microbial infections [36], [37]; in non-infectious diseases, it is thought that extracellular MPO contributes to the majority of unwanted oxidative stress and tissue damage [38], [39]. Thus isolating and assessing extracellular MPO may better reflect induced oxidative damage caused by degranulating myeloid cells and treatment response.

### Validation of ECF Protein Extraction and MPO Retrieval After Protein Precipitation

**Figure 3:**
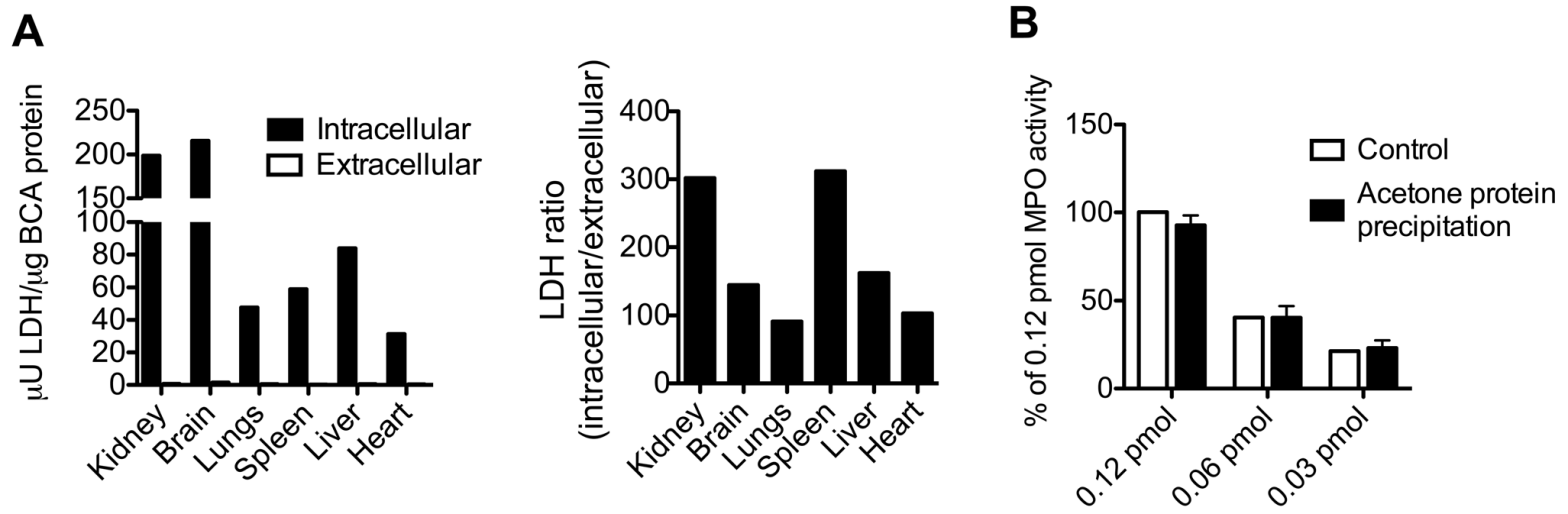
Validation of Extracellular Protein Isolation and MPO Protein Precipitation. (**A**) LDH assay of intra- and extracellular protein fractions of different organs shows that the extracellular fraction only contains very low levels of LDH activity, while the intracellular fraction contains the majority of the LDH activity (left). LDH ratio shows a 90 or higher fold level of ICF LDH over ECF LDH activity (right). (**B**) Protein precipitation of MPO with acetone has no effect on its activity, as evaluated with ADHP (n = 2 per group). LDH = lactate dehydrogenase. BCA = bicinchoninic acid. MPO = myeloperoxidase.

To validate that our method to isolate ECF and ICF works on various organs, we measured the activity of LDH, a strictly intracellular enzyme, on extracts from different organs. The ICF of kidney, brain, lungs, spleen, liver, and heart contained 198.7, 215.6, 47.7, 58.9, 83.9, and 31.3 µU LDH/µg BCA protein, respectively. ECF contained 0.65, 1.48, 0.52, 0.19, 0.52, and 0.30 µU LDH/µg BCA protein, respectively (**Fig. 3a**). The ratio of intra- over extracellular LDH activity normalized to BCA protein was between 91 and 301 (**Fig. 3a**). Taken together, these results suggest that there is no significant contamination from intracellular proteins in our extracellular protein isolation method.

Because of the relatively high volume of extraction buffer needed and the subsequently low protein concentration of the extracellular fluid, it was necessary to concentrate proteins before further use. We tested two methods of protein precipitation: acetone and ammonium sulfate. With ammonium sulfate, the recovered MPO had lost most of its activity (**Fig. S2 in File S1**). In contrast, acetone preserved MPO activity, and over three different concentrations of MPO, we were able to recover 96±5.2% of activity (**Fig. 3b**). This validated acetone protein precipitation as a feasible method to concentrate samples that contain MPO.

### ECF and ICF Protein Extracts Contain Substances that Interfere with MPO Activity Measurements

**Figure 4:**
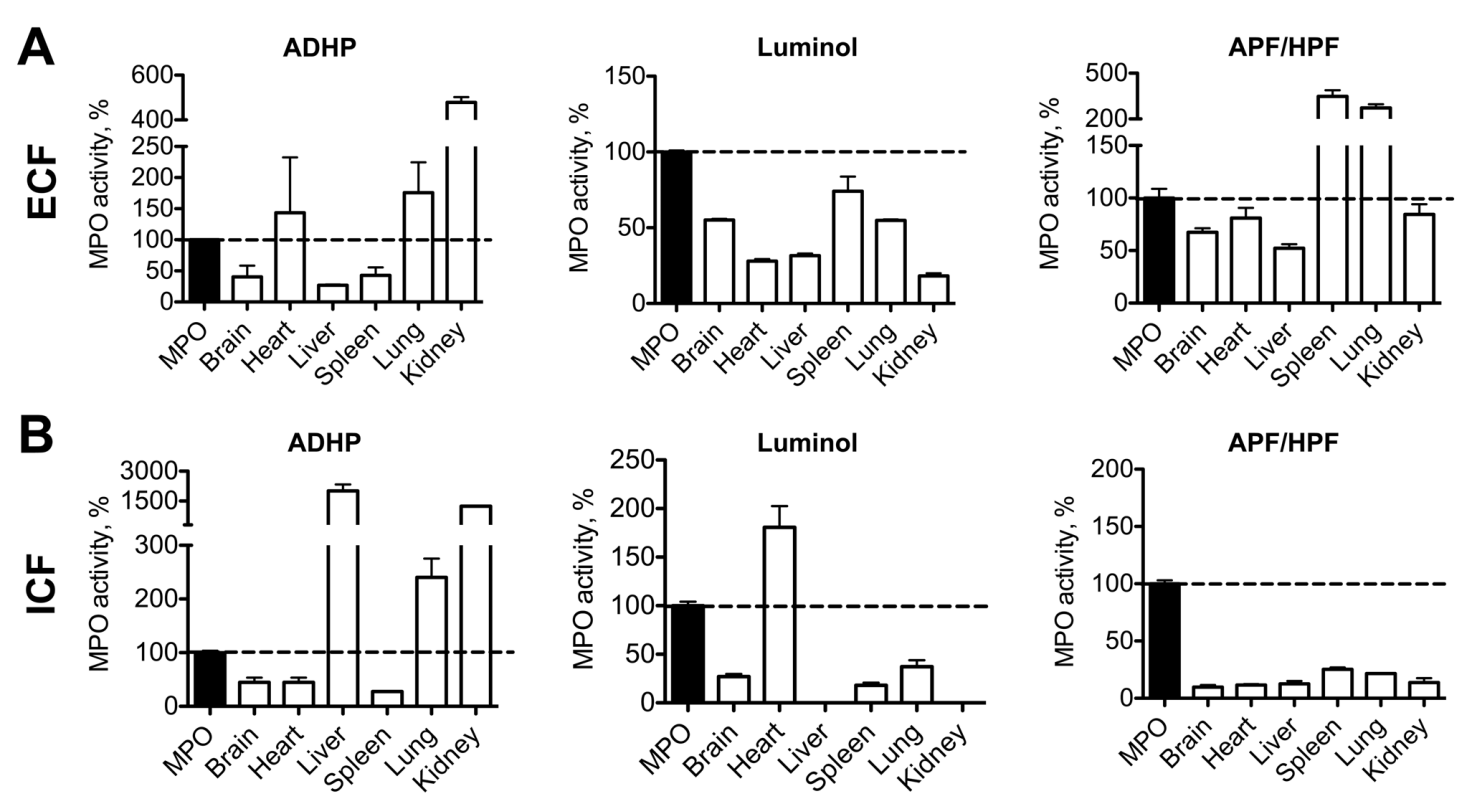
Spike and recovery assay: tissue homogenates and extracellular fluid contain interfering substances. (**A**) Extracellular protein fraction from different organs contains substances interfering with ADHP, luminol, and APF assays (n = 2 per group). (**B**) Intracellular protein fractions also contain interfering substances (n = 2 per group). MPO = myeloperoxidase. ADHP = 10-acetyl-3,7-dihydroxyphenoxazine. APF = 3′-(p-aminophenyl) fluorescein. HPF = 3′-(p-hydroxyphenyl) fluorescein.

To test if our assays could efficiently recover MPO from biological samples, we performed a spike and recovery experiment, where a known amount of human MPO was added to both ECF and ICF extracts from several organs, and MPO activity was measured thereafter. We selected three different MPO assay methods from the literature, which have all been reported to be sensitive and specific to MPO: 1) Bromide dependent chemiluminescence with luminol at acidic pH [29], 2) peroxidase activity with ADHP, and 3) chlorination activity with APF and HPF, where the subtraction of HPF signal from APF signal is thought to represent specific MPO activity [40]. Results were normalized as percentage activity of pure enzyme (**Fig. 4a–b**). For both ECF and ICF, MPO recovery was variable and dependent on the assay and organ used, without a clear recognizable trend. Of note, a large range of MPO activity levels was found, which suggested that peroxidases other than MPO and/or other interfering substances were likely affecting the three assay methods. The nonspecificity of these assays was further confirmed by assaying different concentrations of hemoglobin, which has peroxidase activity [41], with these probes. ADHP and luminol showed a dose-dependent signal increase in these circumstances (**Fig. S3 in File S1**). Based on these findings we conclude that it is necessary to utilize a more specific method for MPO activity detection and hypothesized that antibody-specific binding or extraction of MPO from biological samples before measuring enzyme activity would likely circumvent these issues.

### MPO Antibody Capture Assay is Highly Reproducible

To remove interfering substances from the biological samples being tested, we used an antibody capture assay [42]. First, to establish the reproducibility and linear range of this assay, we loaded homogenized murine neutrophils into anti-MPO antibody-covered wells, and after binding washed away any interfering substances. We then measured MPO activity with ADHP, chosen for its high sensitivity and assay range (**Fig. S4 in File S1**).

For intra-assay reproducibility, triplicates were run simultaneously (**Fig. S5a in File S1**), while for inter-assay reproducibility, each standard curve was run at least 1 hour apart (**Fig. S5b in File S1**). The range of the linear part of the curve was found to be from 598 to 1.2 million neutrophils with coefficients of determination (R^2^) of 0.98 and 0.88 for intra-assay (**Fig. S5a in File S1**, *P*<0.0001) and inter-assay (**Fig. S5b in File S1**, *P*<0.0001) reproducibility, respectively.

This experiment also allowed us to evaluate the sensitivity of this assay, which can detect MPO from as few as 500 neutrophils (**Fig. S5 in File S1**).

### MPO Anti-body Capture Assay Increases the Specificity of MPO Activity Assays in Different Disease Models

**Figure 5:**
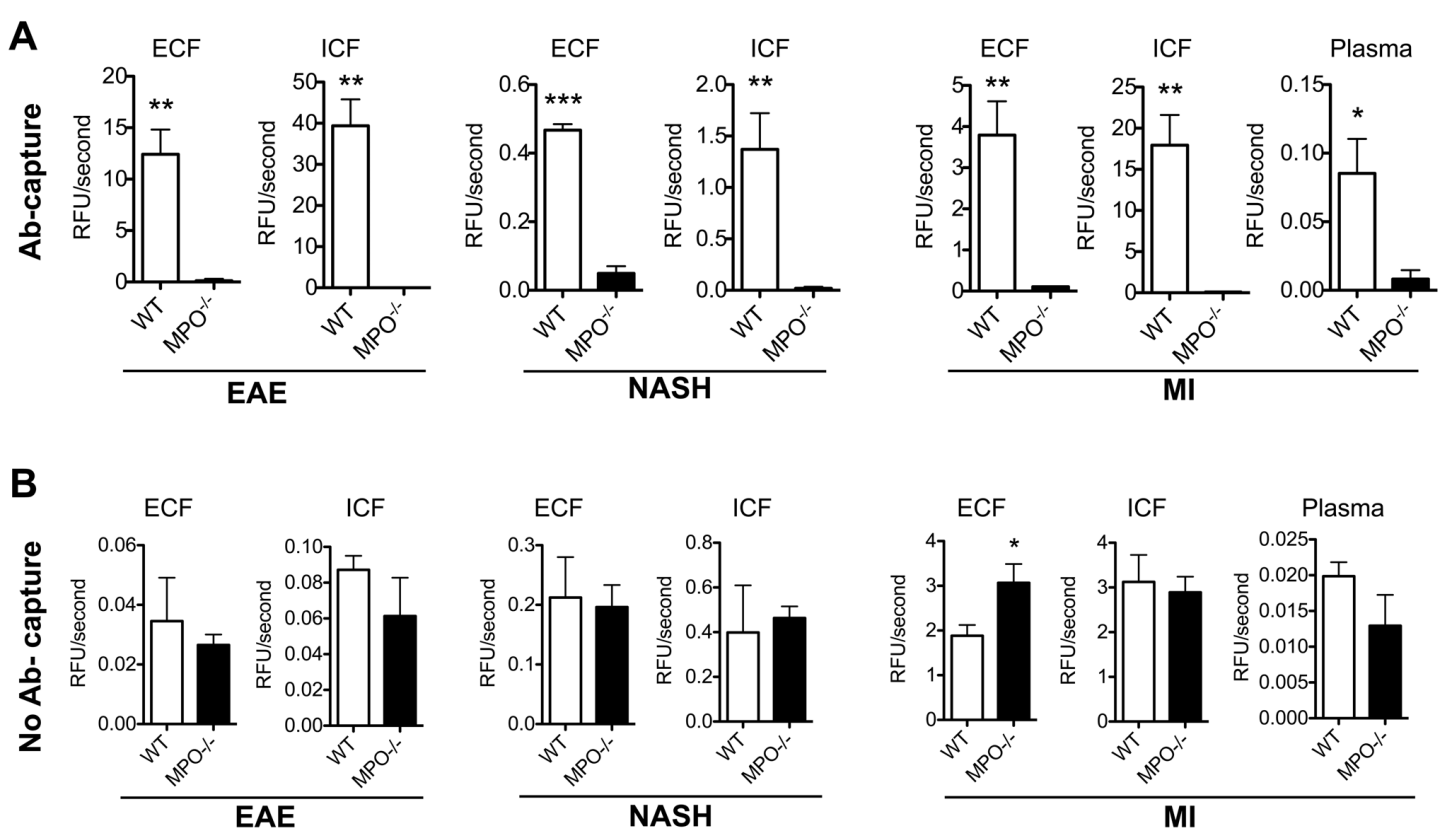
Antibody capture improves the specificity of MPO activity assays on extra- and intracellular extracts in various models of inflammatory diseases. (**A**) Antibody capture of MPO followed by activity detection with ADHP reveals high specificity towards MPO. This is shown in extra- and intracellular fractions in brains from EAE mice, livers from mice with NASH, and hearts and plasma from mice with myocardial infarction (n = 3 per group). (**B**) The same samples processed without antibody capture reveal poor specificity towards MPO, and no significant difference between WT and MPO-KO mice (n = 3 per group). * *P*<0.05. ** *P*<0.01. *** *P*<0.001. ADHP = 10-acetyl-3,7-dihydroxyphenoxazine. MPO = myeloperoxidase. EAE = experimental autoimmune encephalomyelitis. MI = myocardial infarction. NASH = non-alcoholic steatohepatitis. ECF = extracellular fraction. ICF = intracellular fraction. WT = wildtype C57BL/6. MPO^−/−^ = MPO knockout.

**Figure 6:**
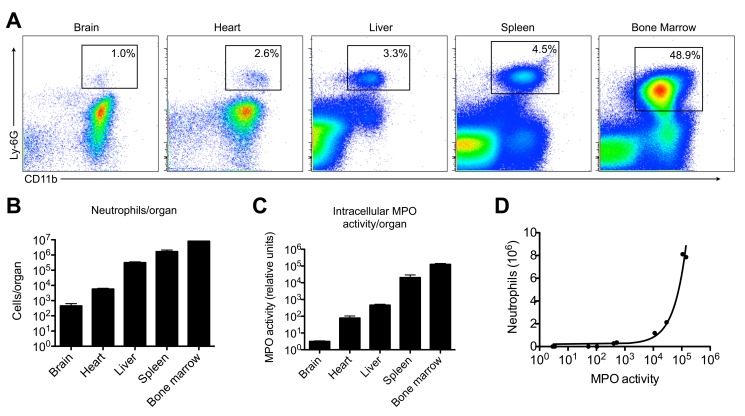
Intracellular MPO activity correlates well with tissue neutrophil content. (**A**) Flow cytometry demonstrates different neutrophil counts in brain, heart, liver, spleen, and bone marrow, as quantified in (**B**) (n = 2 per group). (**C**) Intracellular MPO activity was measured with the antibody-capture assay using ADHP, and shows a similar trend to neutrophil content per organ (n = 2 per group). (**D**) A close correlation was found between neutrophil content and intracellular MPO activity in these organs. MPO = myeloperoxidase. ADHP = 10-acetyl-3,7-dihydroxyphenoxazine.

Next, we validated the specificity of this assay using three different murine inflammatory disease models: 1) EAE, 2) NASH, and 3) MI. We evaluated brains for EAE, livers for NASH, and hearts and plasma for MI in both WT and MPO-KO mice. Both intra- and extracellular extracts were run on the antibody-capture assay with ADHP, as well as with ADHP, luminol, and APF/HPF without antibody-capture. Figure 5 shows results of the ADHP MPO assay, both with (A) and without (B) antibody capture. We detected elevated MPO activity in WT versus MPO-KO mice in ECF fractions (**Fig. 5a**, EAE: 12.41±2.40 vs. 0.12±0.18 RFU/sec, *P* = 0.003; NASH: 0.47±0.02 vs. 0.05±0.02 RFU/sec, *P* = 0.0004; MI: 3.79±0.82 vs. 0.11±0.01 RFU/sec, *P* = 0.006). Consistent with inflammatory cell recruitment in these disease models, we also detected elevated intracellular MPO activity (**Fig. 5a**, EAE: 39.39±6.44 vs. 0.003±0.01RFU/sec, *P* = 0.002; NASH: 1.37±0.35 vs. 0.02±0.01 RFU/sec, *P* = 0.007; MI: 17.94±3.67 vs. 0.09±0.01 RFU/sec, *P* = 0.004). Plasma MPO activity was also elevated in WT versus MPO-KO mice with MI (Fig. 6a, 0.08±0.03, KO: 0.01±0.01 RFU/sec, *P* = 0.02).

In contrast, ADHP without antibody-capture was unable to detect significant differences between WT and MPO-KO homogenates in these three disease models (**Fig. 5b**). In fact, without antibody-capture, the measured MPO activity was higher in the MPO-KO versus WT group in the MI ECF samples (*P* = 0.03). Similar results were obtained with luminol and APF/HPF (**Fig. S6 and S7 in File S1**). This establishes the non-specificity of these assays towards MPO without an antibody-capture step.

### Intracellular MPO Activity Correlates with Tissue Neutrophil Count

Our literature search revealed that the second application of MPO activity assays is to quantify tissue neutrophil content. While extracellular MPO activity is thought to correspond to MPO-induced tissue damage, intracellular activity should correlate with tissue neutrophil numbers. Using flow cytometry to count neutrophils in different organs (**Fig. 6a**), we correlated the results with ICF MPO activity obtained from the same samples. Indeed, we found similar trends between the total numbers of neutrophils per organs (**Fig. 6b**) and ICF MPO activity values (**Fig. 6b**); a very strong correlation was found (R^2^ = 0.966, p<0.0001). These findings indicate that the ICF MPO activity measurement can be used as a surrogate marker for the number of neutrophils contained in a biological sample.

### MPO Activity Assay Protocol

A detailed step-by-step protocol of intra- and extracellular fluid extraction followed by antibody-capture MPO activity assay is given in **File S2**. **Table S1 in File S2** provides troubleshooting for the assay.

## Discussion

Our study showed that the proposed method to isolate intra- and extracellular protein fractions of biological samples is feasible. We found that antibody-capture of MPO is necessary before assessing its activity, due to the non-specificity of available probes and the presence of interfering substances. This method to assay MPO activity was validated in different mouse models of inflammatory conditions and against MPO-KO mice, and a detailed assay protocol is provided.

We selected three probes: ADHP, APF/HPF, and luminol to represent major classes of MPO activity assay probes. ADHP, TMB, *o*-dianisidine, and guaiacol are peroxidase substrates (note that TMB, *o*-dianisidine, and guaiacol are less resistant to autoxidation, are less sensitive, and have a narrower assay range than ADHP [43], [44]; in addition, *o*-dianisidine is carcinogenic [45]). In contrast to these peroxidase probes, APF/HPF can detect the chlorination activity of MPO, and subtracting HPF from APF signal has been suggested to specifically measure HOCl, a highly specific product of MPO [27]. Bromide-dependent chemiluminescence with luminol at low pH has also been shown to be specific towards MPO, and was applied successfully to estimate tumor neutrophil content in a mouse model [28]. Unfortunately, when we tested these assays using samples from multiple different murine organs, we could not adequately recover the spiked MPO activity. Moreover, we did not detect signals in WT mice samples which were substantially greater than those from MPO-KO mice using three different diseases that are known to trigger high tissue levels of MPO. We conclude that interfering substances (e.g. other peroxidases) and tissue inhibitors account for these findings [15], [16].

To circumvent this, we validated an antibody capture assay. In this assay, MPO is bound to the wells of an ELISA plate by means of a monoclonal anti-MPO antibody, which guarantees high specificity. After washing away the unbound substances, enzyme activity can then be detected with a suitable substrate. The capture assay provides researchers with a platform to evaluate both peroxidation and chlorination activity by using different probes without concerns about non-specificity. By using mouse models of myocardial infarction, multiple sclerosis, and steatohepatitis (diseases in which MPO has been implicated in humans), we validated the high specificity and sensitivity of this assay.

In addition to biochemical assays, advanced imaging methods for *in vivo* MPO activity detection are available. These include MPO-Gd, an activatable, ‘smart’ MR imaging probe [22], and the bioluminescent agent luminol [46]. Although both agents have been shown to be highly sensitive and specific *in vivo*, imaging has relatively slow throughput. Thus, a high throughput assay to be used *in vitro* on extracts from biological tissues would be highly desirable to complement the *in vivo* probes.

Another measurement of MPO activity that is widely used is 3-chlorotyrosine, a highly specific product of MPO that can be measured with stable isotope dilution gas chromatography/mass spectrometry [47], [48]. Although specific, 3-chlorotyrosine levels are only a surrogate marker and as such give an estimate of MPO activity in the past, but not necessarily current MPO activity. 3-chlorotyrosine can also be quickly degraded in an inflammatory environment [49], and reactions other than chlorination might be preferentially induced [50]. Thus, its absence does not definitively prove lack of MPO activity. Furthermore, 3-chlorotyrosine levels can be markedly reduced by thiocyanate ions, which are elevated in smokers [51]. All of these findings suggest that 3-chlorotyrosine levels are dependent on the tissue microenvironment, and that direct measurements of MPO activity should be performed whenever possible.

### Conclusions

In summary, we validated a robust protocol to isolate and measure intra- and extracellular MPO activity with high sensitivity and specificity. We validated this assay in three different mouse disease models and in MPO-KO mice. This protocol should be established as the standard method for measuring MPO activity in biological samples. For standardization purposes, we propose the use of ADHP after the antibody capture, due to its wider assay range and higher sensitivity.

## Supporting Information

File S1 **This file contains Figure S1–S7.** Figure S1, Ammonium sulfate protein precipitation decreases recovery of MPO activity. Figure S2, ADHP and luminol assays are sensitive to unspecific peroxidase activity. Figure S3, ADHP is more sensitive and has a wider assay range than APF, TMB, and luminol. Figure S4, Reproducibility of the MPO capture assay. Figure S5, Luminol is not specific for MPO activity in EAE, NASH, or MI. Figure S6, APF/HPF is not specific to MPO in EAE, NASH, or MI. Figure S7, Relative fluorescent units plotted over time with different samples using the antibody-capture MPO activity assay with ADHP.
(PDF)

File S2 **This file contains two items.** Protocol S1, Step-by-Step Protocol. Table S1, Troubleshooting.
(PDF)
