# Supplementary material for: Measuring Myeloperoxidase Activity in Biological Samples
Source: PLoS One. 2013 Jul 5;8(7):e67976. doi: 10.1371/journal.pone.0067976 (PMC3702519; doi:10.1371/journal.pone.0067976)
Supplement: File S1 — This file contains Figure S1–S7. Figure S1, Ammonium sulfate protein precipitation decreases recovery of MPO activity. Figure S2, ADHP and luminol assays are sensitive to unspecific peroxidase activity. Figure S3, ADHP is more sensitive and has a wider assay range than APF, TMB, and luminol. Figure S4, Reproducibility of the MPO capture assay. Figure S5, Luminol is not specific for MPO activity in EAE, NASH, or MI. Figure S6, APF/HPF is not specific to MPO in EAE, NASH, or MI. Figure S7, Relative fluorescent units plotted over time with different samples using the antibody-capture MPO activity assay with ADHP. (PDF) [file pone.0067976.s001.pdf]

Supporting Information File S1

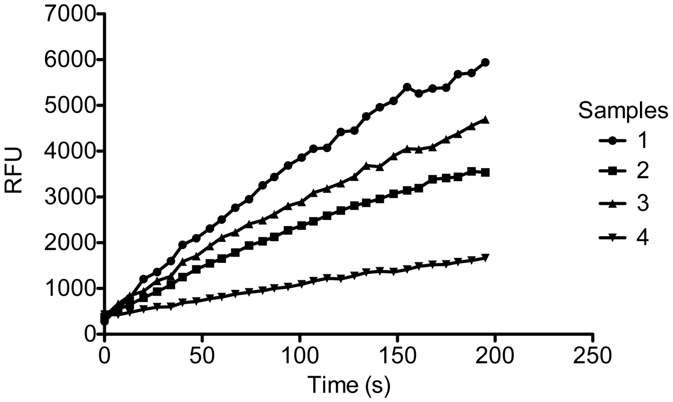

Figure S1: Relative fluorescent units plotted over time with different samples using the antibody-capture MPO activity assay with ADHP.

There is a near-linear increase of ADHP signal in the presence of different MPO concentrations in biological samples.

RFU=relative fluorescence units. ADHP=10-acetyl-3,7-dihydroxyphenoxazine.

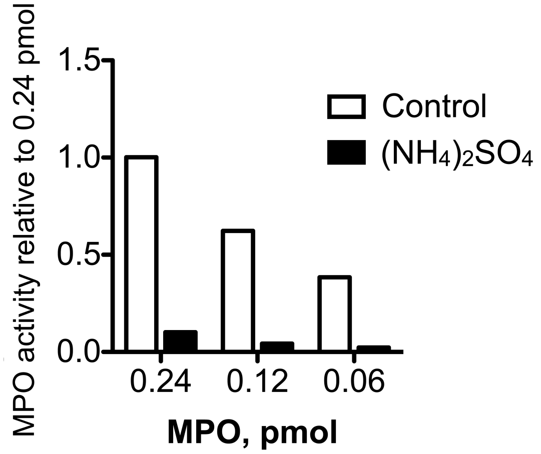

Figure S2: Ammonium sulfate protein precipitation decreases recovery of MPO activity.

MPO was precipitated with $(NH_4)_2SO_4$, and its activity was evaluated. We found significantly decreased MPO activity with ADHP after precipitation with $(NH_4)_2SO_4$ compared to control.

MPO=myeloperoxidase. $(NH_4)_2SO_4$=ammonium sulfate. ADHP=10-acetyl-3,7-dihydroxyphenoxazine.

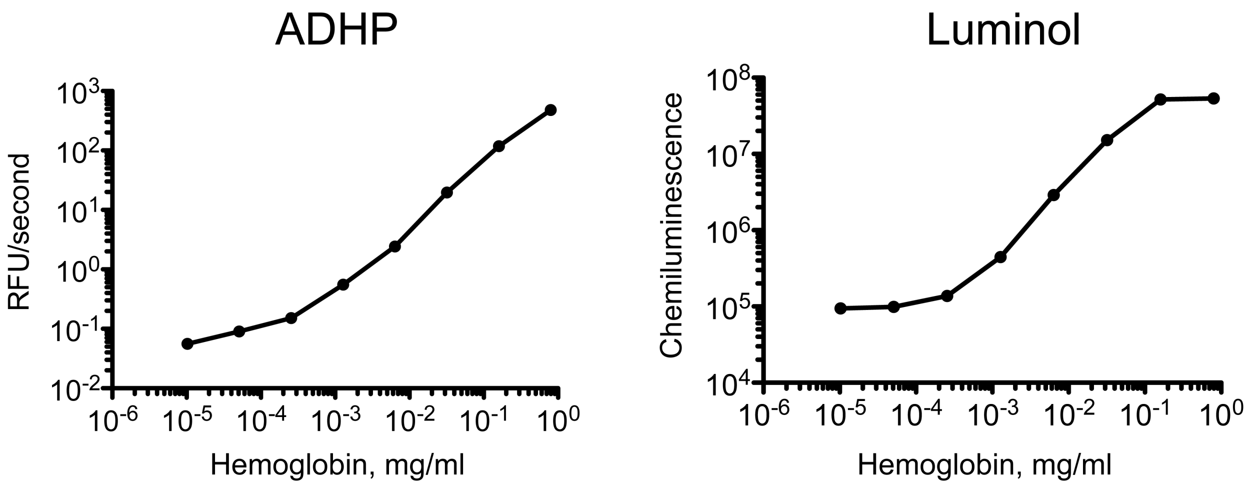

Figure S3: ADHP and luminol assays are sensitive to unspecific peroxidase activity. Increasing concentrations of hemoglobin result in increasing signal in both ADHP and luminol assays. This indicates that both assays are adversely affected by nonspecific peroxidase activity. ADHP=10-acetyl-3,7-dihydroxyphenoxazine.

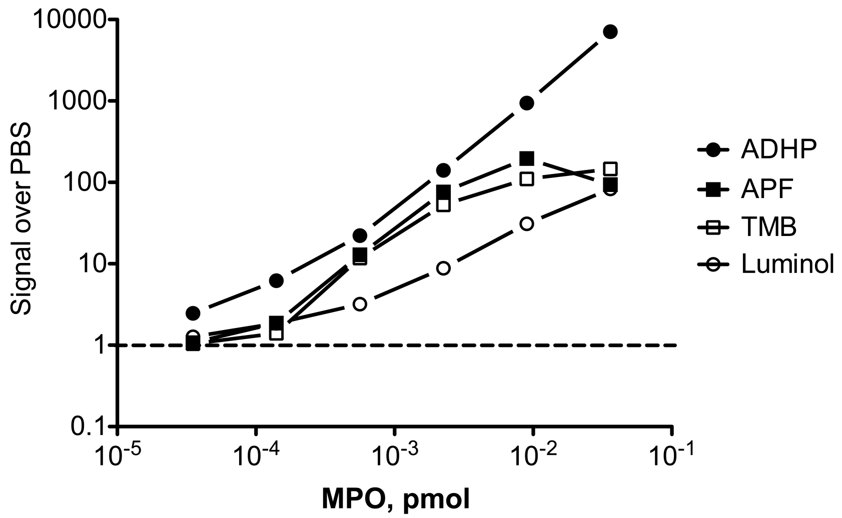

Figure S4: ADHP is more sensitive and has a wider assay range than APF, TMB, and luminol. ADHP is more sensitive, yields consistently higher signal over PBS, and is more linear over the entire range of the MPO standard curve compared to APF, TMB, and luminol.

ADHP=10-acetyl-3,7-dihydroxyphenoxazine. APF=3′-(p-aminophenyl) fluorescein. HPF=3′-(p-hydroxyphenyl) fluorescein. TMB=3,3′,5,5′-Tetramethylbenzidine

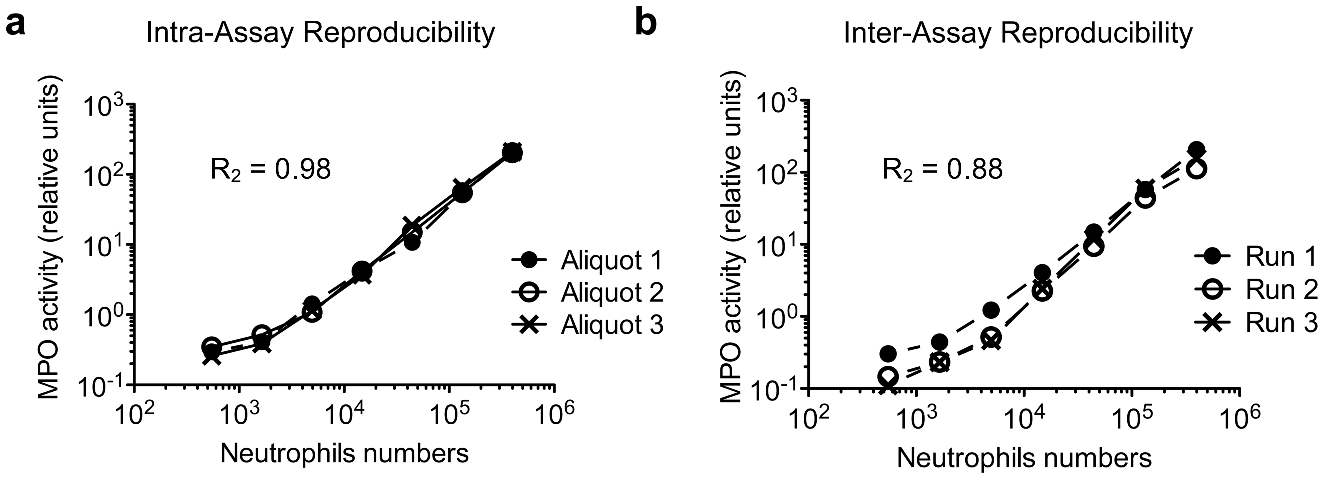

Figure S5: Reproducibility of the MPO capture assay.

(**a**) Intra-assay reproducibility was measured by assaying the same samples in triplicates (coefficient of determination $R_2 = 0.98$, $p<0.0001$). (**b**) Inter-assay reproducibility was measured by performing the assay on the same sample loaded at different times, approximately 1 hour apart . It also shows a strong R_2 value of 0.88 ($p<0.0001$).

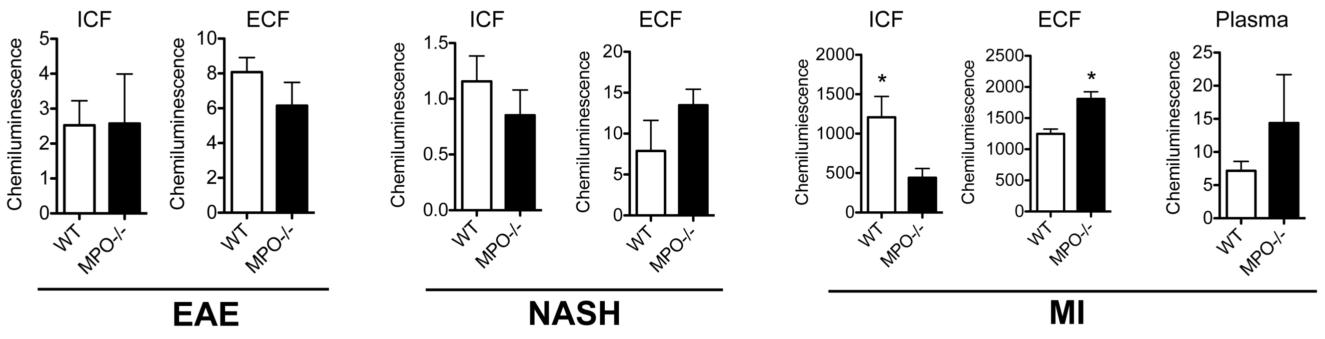

Figure S6: Luminol is not specific for MPO activity in EAE, NASH, or MI.

Poor specificity towards MPO is shown with bromide-dependent chemiluminescence at acidic pH with luminol. In neither EAE nor NASH nor MI could a significant difference between WT and MPO be detected with this assay (n=3 per group).

MPO=myeloperoxidase. EAE=experimental autoimmune encephalomyelitis. MI=myocardial infarction. NASH=non-alcoholic steatohepatitis. ECF=extracellular fraction. ICF=intracellular fraction. WT=wildtype C57BL/6. $MPO^{-/-}$=MPO knockout. * $P < 0.05$.

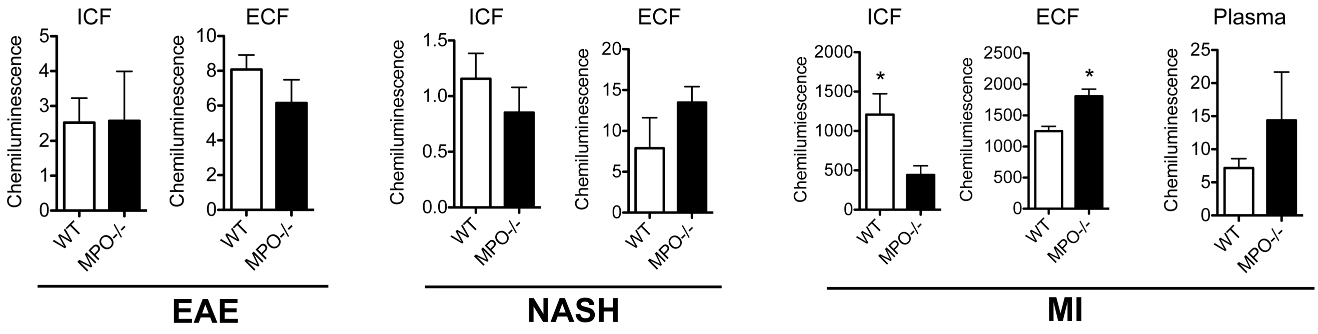

Figure S7: APF/HPF is not specific to MPO in EAE, NASH, or MI.

Poor specificity towards MPO is shown with chlorination detection using APF/HPF. In neither EAE nor NASH nor MI, could a significant difference between WT and MPO be detected with this assay (n=3 per group).

MPO=myeloperoxidase. EAE=experimental autoimmune encephalomyelitis. MI=myocardial infarction. NASH=non-alcoholic steatohepatitis. ECF=extracellular fraction. ICF=intracellular fraction. WT=wildtype C57BL/6. $MPO^{-/-}$=MPO knockout. APF=3′-(p-aminophenyl) fluorescein. HPF=3′-(p-hydroxyphenyl) fluorescein. * $P < 0.05$.
