# Supplementary material for: Measuring Myeloperoxidase Activity in Biological Samples
Source: PLoS One. 2013 Jul 5;8(7):e67976. doi: 10.1371/journal.pone.0067976 (PMC3702519; doi:10.1371/journal.pone.0067976)
Supplement: File S2 — This file contains two items. Protocol S1, Step-by-Step Protocol. Table S1, Troubleshooting. (PDF) [file pone.0067976.s002.pdf]

Supporting Information File S2

Step-by-Step Protocol

Reagents:

- Sucrose (Sigma, S3089)
- $CaCl_2$ (Sigma, C5770)
- Heparin Sodium 1000 USP Units/mL (APP Pharmaceuticals, Schaumburg, IL)
- Phosphate-buffered Saline (Boston Bioproducts, BM-220S)
- Acetone (Fisher Scientific, S70091)
- Cetyltrimethylammonium bromide (Sigma, H6269)
- Potassium phosphate monobasic (Sigma, P9791)
- Potassium phosphate dibasic (Sigma, P3786)
- Tween-20 (Sigma, P1379)
- Amplite ADHP (10-Acetyl-3,7-dihydroxyphenoxazine) (AAT Bioquest, 11000)
- Hydrogen peroxide (Sigma, 323381)
- Hanks balanced salt solution (HBSS, Lonza, 10-547F)
- Dimethyl sulfoxide (DMSO, Sigma, D4540)
- Isoflurane (Forane, Baxter)
- MPO ELISA dilution buffer (Hycult, HK210-02)

Equipment

- Freezer (−80°C and −20°C) and 4°C refrigerator
- 15 ml conical tubes (BD 352097) and 50 ml conical tubes (BD 352098)
- 1.5 ml tubes (Eppendorf, 3810X)
- 2 ml round bottom tubes (Corning, 431386)
- Plate reader capable of excitation wavelength of 535 nm and emission wavelength of 590 nm (Tecan Safire 2 or comparable)
- Ultracentrifuge capable of holding 50 ml conical tubes and centrifugation speed of 3,500 g (Fisher Scientific Sorvall RC 6 Plus)
- (Alternative: 50 kD molecular weight cutoff filter tubes, Millipore, UFC805096)
- Harvard Apparatus 11 Plus Syringe Pump (Harvard Apparatus, 702208) or similar
- 20 ml syringe (BD 309661)

- Tissue homogenizer (Tissuemiser, Fisher Scientific, 15-338-420)
- Pipette-Aid (Drummond, 4-000-101)
- 10 ml glass pipettes (VWR, 93000-680)
- Bicinchoninic acid protein assay (BCA assay, Thermo Scientific, 23227)
- GraphPad Prism, Excel, or other software capable of performing a linear regression
- MPO ELISA plates (Hycult, HK210-02)
- Isoflurane vaporizer (Colonial Medical Supply, VIP 3000 or comparable)

Reagent Setup:

- **CTAB buffer** (50 mM potassium phosphate at pH 6.0 with 50 mM CTAB, can be stored at room temperature (RT) for up to 1 year)
- **Extraction buffer** (0.32 M sucrose, 1 mM $CaCl_2$, 10U/ml Heparin in HBSS, can be stored at RT for up to 1 year)
- **ADHP stock solution** (200 mM ADHP in DMSO, 15 µl aliquots can be stored at -20°C for up to 1 year)
- **ADHP working solution** (prepare right before assay, dilute ADHP stock solution 1:1000 in PBS)
- **H_2O_2 working solution** (prepare right before assay, dilute 3% H_2O_2 1:100 in PBS)
- **Washing buffer** (PBS with 0.05% Tween-20)

Protocol:

Extracellular protein extraction (Timing: 3 hours)

1. Equilibrate acetone (25 times the weight of each sample) at -20°C.
2. Anesthetize mouse with 1-3% isoflurane in O_2.
   CAUTION: Check proper depth of anesthesia (e.g. by checking reflexes and/or breathing pattern before continuing to next step).
3. Perform a cardiac perfusion with 20 ml ice-cold PBS at 2-5 ml per minute using a perfusion pump, harvest organ of interest.
   CAUTION: Ensure that this method of euthanasia is approved on your IACUC protocol.
3. Wash organ of interest briefly in PBS in a petri dish.
4. In a 15 ml conical tube, incubate organ for 2 hours on ice (or 0°C) in 4 times the organ weight in **extraction buffer** to extract extracellular proteins

CRITICAL STEP: Use fresh ice or a 0°C chamber to minimize cell death and avoid leakage of intracellular proteins.

5. Transfer organs into 2 ml round bottom tubes, store on ice (will be used to extract intracellular protein, see step 12)
6. Centrifuge extraction buffer containing extracellular proteins for 5min at 500 g at 4°C. This ensures absence of loose tissue debris and cells from the extracellular fraction. Then transfer the supernatant to 50 ml conical tubes.
7. Slowly add 4 times the supernatant volume of cold acetone (from step 1) to the tube, incubate for 1 hour at -20°C to precipitate extracellular proteins.

   CAUTION: Acetone is a good solvent for polystyrene, polycarbonate, and polypropylene, and will dissolve pipettes made from these materials. Use 10ml glass pipettes instead.
8. Centrifuge at 3,500 g for 15 minutes at 4°C, then decant supernatant completely.

   CAUTION: Before centrifugation, mark the tubes on the centrifugal side with a permanent marker, this will make the protein pellet easier to identify after centrifugation.
9. Air-dry precipitated proteins for 5-10 minutes

   CRITICAL STEP: Acetone needs to completely evaporate for a successful resuspension, but an overdried pellet will also be hard to resuspend. In our experience, 5-10 minutes drying time works best (see Table 2).
10. Resuspend protein pellet in 0.3 ml of PBS by using a 200 µl pipette. Repeatedly pipette PBS up and down where the protein pellet is visualized without creating bubbles. Do not vortex the solution because MPO may be sensitive to vigorous vortexing.

    ALTERNATIVE: Instead of acetone protein precipitation, extracellular proteins can also be concentrated by using 50 kD MWCO filter tubes according to manufacturer's instructions (instead of steps 7-10).

Intracellular protein extraction (Timing: 30 minutes)

11. Add 500 µl of **CTAB buffer** to organs from step 6; homogenize organs for 30 seconds using a mechanical tissue homogenizer, and transfer homogenate to 1.5 ml tubes.
12. Sonicate for 30 seconds in a water-bath sonicator, freeze-thaw samples in liquid nitrogen.
13. Centrifuge at 15,000 g for 15 minutes at 4°C.
14. Collect supernatant (= intracellular proteins).

Antibody capture MPO activity assay (Timing: 2 hours)

Pause point: Samples can be stored at -80°C for up to 3 months at this point.

15. Perform BCA assay according to manufacturer's instructions to obtain protein concentration per sample.

    CAUTION: Proceed directly to MPO activity assay after performing BCA protein determination to ensure best results.

16. Dilute samples from steps 11 and/or 15 1:2 to 1:20 in dilution buffer (Hycult), depending on the expected activity, or load a certain amount of protein as determined by BCA assay.
17. Add diluted samples to MPO activity assay plates (Hycult), which have MPO-antibody coated on well bottom.

    CAUTION: Always Include a negative control (PBS only), and a positive control (e.g., isolated bone-marrow derived neutrophils, a detailed protocol can be found in the methods section [30]) in the experimental layout.

18. Add 100 µl of diluted samples per well, incubate for 1 hour at room temperature.
19. Remove samples from wells, wash wells 4 times with 300µl **washing buffer**.
20. Add 49 µl PBS + 1 µl **H_2O_2 working solution** to each well
21. Add 50 µl **ADHP working solution** to each well.

    CRITICAL STEP: Add **H_2O_2 working solution** and **ADHP working solution** in rapid succession, and immediately acquire data without delay. In the presence of H_2O_2, MPO will start to convert ADHP to the fluorescent resorufin.

    ALTERNATIVE: Instead of ADHP, other probes such as TMB, luminol, or APF/HPF can be used. In our experience, however, ADHP is more sensitive and reliable than TMB, and plate readers capable of detecting chemiluminescence of luminol are not as widely available.

22. Acquire fluorescence in kinetic mode for 5 to 10 minutes (excitation 535 nm, emission 590 nm); a linear increase should be observed (see **Fig. S1 in File S1**).
23. OPTIONAL: Wells can be washed 4 times with **washing buffer**, and specific MPO activity (RFU/mg MPO protein) can be measured by continuing the ELISA as per manufacturer's instructions.

Data analysis (Timing: 30 minutes)

24. Subtract values from PBS control (ADHP may be activated by H_2O_2 alone, which has to be accounted for).
25. Calculate slope of all cycles by using a linear regression (e.g. in Prism) or the slope function in Excel [slope (sample values, time in seconds]. The slope represents the enzyme activity.
26. Normalize activity for sample volume, dilution, and/or protein concentration. Results can then be reported as RFU per second.

Table S1: Troubleshooting

Step	Problem	Possible Reason	Solution
3	Blood remains in organ	Poor perfusion technique	Make sure to cannulate left ventricle of the heart only once with a 25 G needle.<br>Increase volume of perfusate.
5	Intracellular proteins found in extracellular fluid	Cell death	Use fresh ice and add more ice during the incubation time.<br>Bury tubes in fresh ice during incubation time.
8	No protein pellet visible	Pellet is small (e.g. because of low protein concentration)<br><br>Pellet is absent	Mark centrifugal side of the tube before centrifugation, this will make identification of the pellet easier.<br><br>Increase centrifugation speed and/or time.
10	Dried pellet does not go into solution	Underdried pellet<br><br>Overdried pellet<br><br>Poor manual resuspension	Increase drying time.<br><br>Decrease drying time.<br><br>Repeatedly pipette PBS up and down, against the protein pellet.<br>Consider using MWCU filters to avoid this problem.
14	No clear supernatant visible	Sample not fully homogenized<br><br>Organs with high-lipid content (e.g. brain)	Increase mechanical homogenization time.<br><br>Increase centrifugation time. Perform a second spin to reduce lipid content.

26	Low MPO activity	Repeated freeze-thaw cycles of samples.	Avoid more than one freeze-thaw cycle per sample, do BCA and MPO activity assay at the same time.
		Poor antibody binding	Increase incubation time to 2 hours.
		Dilution too high	Try lower dilution of samples. For extracellular fraction samples, resuspension in less PBS after acetone precipitation may help.
	RFU slope is not linear	MPO concentration is too high	Increase dilution of samples.
		Delay between addition of ADHP and acquisition	Immediately start acquiring data after adding ADHP and H_2O_2.
	No MPO activity in neutrophil standards	Insufficient number of neutrophils isolated	Carefully count neutrophils before homogenization.
		Isolation of neutrophils unsuccessful	Confirm success of neutrophil isolation using flow cytometry or immunocytochemistry for MPO.
